# Blood-based protein profiling identifies serum protein c-KIT as a novel biomarker for hypertrophic cardiomyopathy

**DOI:** 10.1038/s41598-020-80868-z

**Published:** 2021-01-19

**Authors:** Kristina Sonnenschein, Jan Fiedler, David de Gonzalo-Calvo, Ke Xiao, Angelika Pfanne, Annette Just, Carolin Zwadlo, Samira Soltani, Udo Bavendiek, Theresia Kraft, Cristobal Dos Remedios, Serghei Cebotari, Johann Bauersachs, Thomas Thum

**Affiliations:** 1 Institute of Molecular and Translational Therapeutic Strategies (IMTTS), Hannover Medical School, Carl-Neuberg-Strasse 1, 30625 Hannover, Germany; 2 Department of Cardiology and Angiology, Hannover Medical School, Hannover, Germany; 3 CIBER of Respiratory Diseases (CIBERES), Institute of Health Carlos III, Av. de Monforte de Lemos, 28029 Madrid, Spain; 4 Translational Research in Respiratory Medicine, IRBLleida, University Hospital Arnau de Vilanova and Santa Maria, Av. Alcalde Rovira Roure 80, 25198 Lleida, Spain; 5 Institute of Molecular and Cell Physiology, Hannover Medical School, Hannover, Germany; 6 Anatomy and Histology, School of Medical Sciences, Bosch Institute, University of Sydney, Camperdown, Australia; 7 Department of Cardiac, Thoracic, Transplantation, and Vascular Surgery, Hannover Medical School, Hannover, Germany; 8 REBIRTH Center for Translational Regenerative Medicine, Hannover Medical School, Hannover, Germany; 9 Fraunhofer Institute of Toxicology and Experimental Medicine, Hannover, Germany

**Keywords:** Biomarkers, Cardiology, Medical research

## Abstract

Hypertrophic cardiomyopathy (HCM) is one of the most common hereditary heart diseases and can be classified into an obstructive (HOCM) and non-obstructive (HNCM) form. Major characteristics for HCM are the hypertrophy of cardiomyocytes and development of cardiac fibrosis. Patients with HCM have a higher risk for sudden cardiac death compared to a healthy population. In the present study, we investigated the abundancy of selected proteins as potential biomarkers in patients with HCM. We included 60 patients with HCM and 28 healthy controls and quantitatively measured the rate of a set of 92 proteins already known to be associated with cardiometabolic processes via protein screening using the proximity extension assay technology in a subgroup of these patients (20 HCM and 10 healthy controls). After validation of four hits in the whole cohort of patients consisting of 88 individuals (60 HCM patients, 28 healthy controls) we found only one candidate, c-KIT, which was regulated significantly different between HCM patients and healthy controls and thus was chosen for further analyses. c-KIT is a tyrosine-protein kinase acting as receptor for the stem cell factor and activating several pathways essential for cell proliferation and survival, hematopoiesis, gametogenesis and melanogenesis. Serum protein levels of c-KIT were significantly lower in patients with HCM than in healthy controls, even after adjusting for confounding factors age and sex. In addition, c-KIT levels in human cardiac tissue of patients with HOCM were significant higher compared to controls indicating high levels of c-KIT in fibrotic myocardium. Furthermore, c-KIT concentration in serum significantly correlated with left ventricular end-diastolic diameter in HOCM, but not HCM patients. The present data suggest c-KIT as a novel biomarker differentiating between patients with HCM and healthy population and might provide further functional insights into fibrosis-related processes of HOCM.

## Introduction

Hypertrophic cardiomyopathy (HCM) is a common inherited cardiovascular disease caused by gene mutations mainly in cardiac sarcomere proteins^1^. HCM is subdivided into obstructive (HOCM) and non-obstructive (HNCM) form and is characterized by hypertrophy of cardiomyocytes as well as ventricular fibrosis. Clinical manifestation of hypertrophic cardiomyopathy is variable and ranges from asymptomatic forms to terminal heart failure or sudden cardiac death^2^. Even within the disease itself the symptoms and therapy in patients with HOCM and HNCM are partially different. Unlike HNCM, HOCM develops a pathological gradient in the left ventricular outflow tract causing an increased systolic flow rate ultimately leading to a higher degree of mitral valve insufficiency. Therefore further interventional or surgical therapeutic options besides drug therapy are available for HOCM patients. Despite the high prevalence of HCM with 1:500 it is often under-diagnosed and therefore biomarkers are needed to detect this detrimental disease at an early stage and manage the clinical symptoms of patients with unidentified HCM.

Several strategies to explore biomarkers have been pursued in the last years and provided information about circulating long non-coding RNAs^3–5^, microRNAs^6,7^, positron emission tomography (PET)^8^ or cardiac MRI parameters^9^ as well as circulating natriuretic peptides^10^ involved into pathophysiology of HCM. First insights into proteomic data from myocardial hypertrophic tissues have been recently provided and several dysregulated proteins in septal myectomies in patients with HCM were described^11^. Nevertheless, detailed correlations to the circulating proteins in blood of HCM patients are still rare.

Reduction in the efficiency of cardiometabolic processes in HCM has been recently characterized^12,13^ leading to impaired sarcomere energetics including altered sarcomere activity with lowered cardiac contractility^14^. In the present study we analyzed a series of circulating proteins known to be involved in cardiometabolic processes such as cell adhesion, immune response, complement activation and cellular metabolism, to identify circulating target proteins which were then correlated with tissue levels in human septal myectomies.

c-KIT, also known as tyrosine-protein kinase KIT, mast/stem cell growth factor receptor (SCFR) or CD117, is a plasma protein and receptor for the stem cell factor (SCF). It is mainly localized to the plasma membrane of different cell types, e.g. endothelial cells, fibroblasts or hematopoietic cells, in tissues as well as in body fluids. One of the possible mechanisms for the generation of soluble form of c-KIT is a metalloproteolytic octodomain shedding of c-KIT and it was already described in fibroblasts and mast cells^15^. Binding of SCF to c-KIT leads to an activation of several pathways (e.g. JAK/STAT3 or PI3K/AKT pathways) regulating amongst others cell proliferation and survival, hematopoiesis, gametogenesis and melanogenesis^16^. c-KIT mutations are associated with gastrointestinal, dermatological and hematological tumors^17–21^ and c-KIT acts as a biomarker in different cancer types^22,23^. c-KIT expression has also been described in the detection and assessment of the prognosis of patients with tumors^24,25^. Recently the role of c-KIT in the development of bleomycin-induced pulmonary fibrosis was described by Ding et al.^26^. Investigating the importance of cardiac c-KIT has become the aim of the present study. Here, we identified serum protein c-KIT as a potential biomarker for HCM distinguishing between patients with hypertrophic cardiomyopathy and healthy subjects.

## Results

We included 60 age- and sex-matched patients with HCM and 28 healthy control individuals to the present study. 33 patients among the HCM cohort had an obstruction (HOCM) and 27 were without obstruction (HNCM) in the left ventricular outflow tract. Detailed patients’ characteristics are shown in Table 1. Patients were chosen according to the diagnostic criteria based on the recent European guidelines for the diagnosis and management of hypertrophic cardiomyopathies^2^.

**Table 1:** Characteristics of the study population.

Variable	Control	HCM	Control:HCM	HNCM	HOCM	HNCM:HOCM
	n = 28	n = 60		n = 33	n = 27	
	n (%) / median (P25-P75)	n (%) / median (P25-P75)	*p* value	n (%) / median (P25-P75)	n (%) / median (P25-P75)	*p* value
Age (years)	52 (44.3–59.5)	52.5 (42.8–65.0)	0.628	50 (34.5–61.0)	60.0 (47.0–73.0)	0.047
Male n (%)	19 (67.9)	39 (65.0)	1.000	25 (75.8)	14 (51.9)	0.063
Body mass index (kg/m^2^)		27.0 (24.5–30.8)	NA	26.9 (24.1–30–7)	27.3 (24.5–31.1)	0.513
**Echocardiographic parameters**						
IVS (mm)		18.5 (16.0–22.0)	NA	17.0 (15.0–22.0)	19.0 (17.0–23.0)	0.192
LVEDD (mm)		43.0 (40.0–47.3)	NA	43.0 (41.0–48.0)	42–0 (38.0–47.0)	0.192
Aortic root (mm)		32.0 (28.5–35.0)	NA	32.0 (27.0–35.0)	32.0 (28.8–35.3)	0.520
LVPWD (mm)		12.0 (9.9–13.3)	NA	11.0 (9.5–12.0)	12.0 (10.0–15.0)	0.105
LVOT gradient (mmHg)		80.0 (13.5–121.5)	NA	7.6 (5.5–15.0)	103.0 (80.0–140.0)	< 0.001*
LA size (mm)		42.0 (38.0–50.5)	NA	40.0 (35.0–49–0)	44.0 (40. –51.3)	< 0.001*
**Mitral regurgitation**						0.001
Minor		40 (66.7)		28 (84.8)	12 (44.4)	
Medium		13 (21.7)		3 (9.1)	10 (37.0)	
Major		5 (8.3)		0 (0.0)	5 (18.5)	
Missing		2 (3.3)		2 (6.1)	0 (0.0)	
**Clinical symptoms**						
Syncope n (%)		8 (13.8)	NA	3 (9.7)	5 (18.5)	0.453
Family history for SCD n (%)		18 (30.5)	NA	12 (37.5)	6 (22.2)	0.262
Dyspnoea n (%)		32 (54.2)	NA	14 (43.8)	18 (66.7)	0.116
**NYHA n** (**%)**						0.103
1		12 (20.0)		9 (27.3)	3 (11.1)	
2		27 (45.0		11 (33.3)	16 (59.3)	
3		13 (21.7)		5 (15.2)	8 (29.6)	
Missing		8 (13.3)		8 /24.2)	0 (0.0)	
Angina pectoris n (%)		9 (153)	NA	2 (6.3)	7 (25.9)	0.066
Palpitations n (%)		22 (37.3)	NA	11 (34.3)	11 (40.7)	0.788
Peripheral edema n (%)		4 (6.9)	NA	2 (6.1)	2 (7.4)	1.000
Arrhythmias n (%)		31 (52.5)	NA	19 (59.4)	12 (44.4)	0.302
Atrial Fibrillation n (%)		5 (8.6)	NA	4 (12.1)	1 (3.8)	0.367
**Co-morbidities**						
Hypertension n (%)		30 (51.7)	NA	17 (54.8)	13 (48.1)	0.793
Diabetes mellitus n (%)		5 (8.6)	NA	3 (9.7)	2 (7.4)	1.000
Coronary artery disease n (%)		12 (20.7)	NA	5 (16.1)	7 (25.9)	0.518
Myocardial infarction n (%)		4 (7.0)	NA	2 (6.1)	2 (7.7)	1.000
COPD n (%)		3 (5.2)	NA	0 (0.0)	3 (11.1)	0.095
**Drugs**						
Beta blockers n (%)		46. (78.0)	NA	24 (75.0)	22 (81.5)	0.754
ACE inhibitors n (%)		17 (28.8)	NA	10 (31.3)	7 (25.9)	0.776
AT1 inhibitors n (%)		10 (16.9)	NA	9 (28.1)	1 (3.7)	0.016*
Diuretics n (%)		24 (40.7)	NA	11 (34.4)	13 (48.1)	0.303
Calcium antagonists n (%)		17 (28.8)	NA	8 (25.0)	9 (33.3)	0.569
Anticoagulation drugs n (%)		30 (50.8)	NA	15 (46.9)	15 (55.6)	0.604

*HCM* hypertrophic cardiomyopathy, *HNCM* non-obstructive hypertrophic cardiomyopathy, *HOCM* hypertrophic obstructive cardiomyopathy, *NA* not applicable, *IVS* interventricular septum size, *LVEDD* left ventricular end-diastolic diameter, *LVPWD* left ventricular posterior wall thickness end diastole, *LVOT* gradient = left ventricular outflow tract gradient maximum, *LA* left atrium, *AT1* angiotensin II receptor antagonist, *ACE* angiotensin-converting enzyme, *COPD* chronic obstructive pulmonary disease, *SCD* sudden cardiac death.

Data are presented as frequencies (percentages) for categorical variables. Continuous variables are presented as median (P25-P75). Differences between study groups were analysed using Mann–Whitney U test, Fisher's exact test or chi-squared test.

* Statistically significant.

The patients in the HNCM and HOCM group did not differ in clinical symptoms, NYHA classification and co-morbidities. Differences were found in the medication with higher use of AT1 receptor antagonists in HNCM patients, more severe grade mitral regurgitation in HOCM group and in the echocardiographic parameters according to the size of left atrium with larger atrium in the HOCM group. There were no differences in the dimensions of the septum thickness between HNCM and HOCM as well as in the left ventricular end-diastolic diameters. Due to the pathological consequences of the obstructive form of HCM the maximum gradient in the left ventricular outflow tract was higher in the HOCM group.

**Figure 1:**
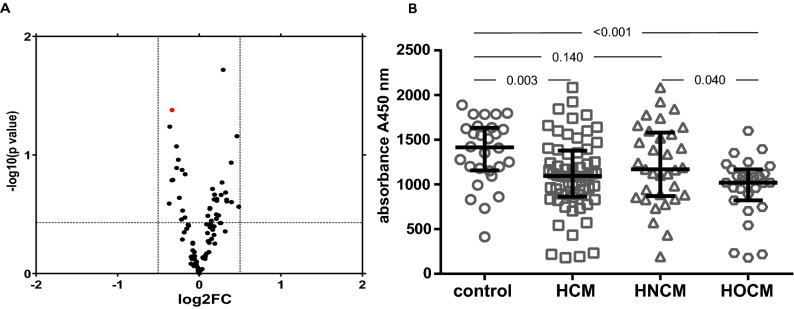
Proteomic approach of cardiometabolic panel of proteins was performed in serum of patients in the study groups and subsequently validated by ELISA assay. (**A**) Proteomic measurement. n = 10 in each group. c-KIT is labeled in red. (**B**) ELISA assay for c-KIT. n = 27–33 in each group. *p* values describe the significance level of differences for each comparison. *HCM* hypertrophic cardiomyopathy, *HNCM* non-obstructive hypertrophic cardiomyopathy, *HOCM* obstructive hypertrophic cardiomyopathy.

**Figure 2:**
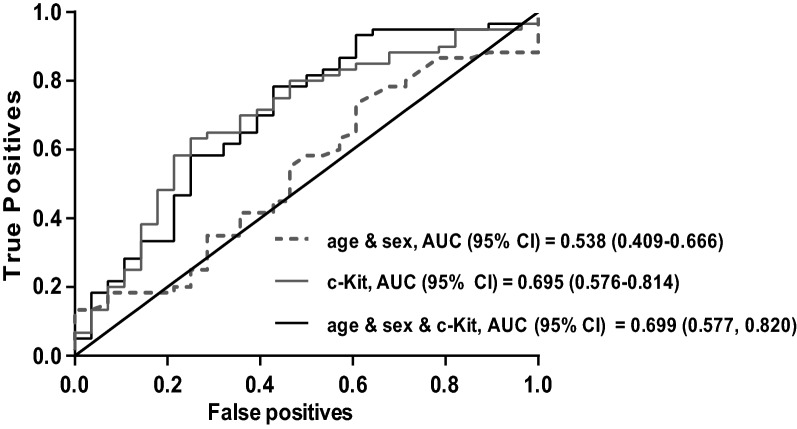
ROC curve analyses in controls versus HCM patients. Data are presented as the area under the ROC curve (AUC) and 95% confidence intervals (CI). N = 27–33 in each group. *HCM* hypertrophic cardiomyopathy.

We applied a proteomic profiling approach by proximity extension assay (PEA) technology to find potential protein biomarkers in the serum of total 30 patients (10 patients with HOCM, 10 patients with HNCM and 10 healthy individuals) (Fig. 1A). We screened for 92 proteins known to be involved in several cardiometabolic processes including cell regulation, angiogenesis, apoptosis, immune response and heart development and came down to four significantly regulated candidates (c-KIT, IL7R, PCPE-1 and IGLC2), which were then subsequently validated via ELISA assay in a larger cohort of patients’ sera (60 HCM, 28 healthy). The protein level of tyrosine-protein kinase c-KIT was significantly lower in patients with HCM than in healthy controls, whereby a significant difference occurred between HOCM and HNCM patients (Fig. 1B). Adjustment for age and sex did not affect this association between c-KIT and HCM showing that c-KIT is independently associated with HCM (Table 2). c-KIT was inversely associated with the presence of HCM. Interestingly, the area under the curve (AUC) for c-KIT alone was higher than for age and sex together (AUC 0.695 vs 0.538) (Fig. 2).

**Table 2:** Association between c-Kit and HCM.

Model	Variable	OR (95% CI)	*p* value
Model 1	c-Kit	0.998 (0.997–0.999)	0.009
Model 2	c-Kit	0.998 (0.997–0.999)	0.006
	Age	0.982 (0.946–1.020)	0.348
	Sex	0.924 (0.337–2.538)	0.879

*OR* Odd ratio, *95% CI* 95% confidence interval.

Model 1 is an unadjusted Model. Model 2 is adjusted for age and sex. OR Odds ratio, 95% CI 95% confidence interval.

**Figure 3:**
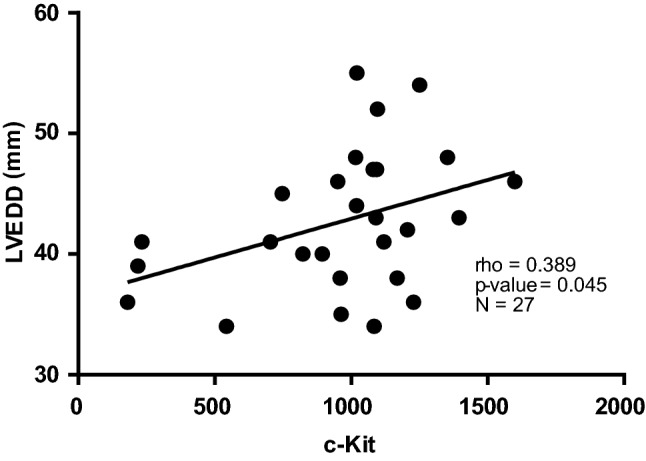
Correlations between c-KIT levels and echocardiographic parameters in patients with obstructive hypertrophic cardiomyopathy (HOCM). c-KIT vs LVEDD in patients with HOCM. n = 27–33 in each group. Correlations between variables were analyzed using Spearman’s rho coefficient. LVEDD: left ventricular end-diastolic diameter.

Furthermore, we performed correlation analyses of a potential association of HCM with echocardiographic parameters. There were no correlations between c-KIT and echocardiographic parameters in the whole HCM cohort. However, we found a direct significant correlation between c-KIT and left end-diastolic diameter (LVEDD) in HOCM but not HNCM patients (Fig. 3).

**Figure 4:**
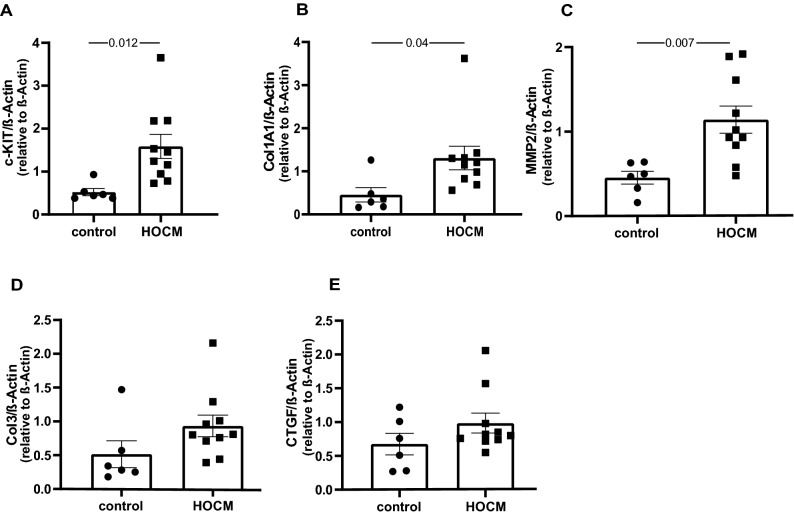
Measurement of c-KIT levels in human biopsies and correlation with pro-fibrotic markers in obstructive hypertrophic cardiomyopathy (HOCM) via quantitative Real-time PCR. (**A**) c-KIT levels in HOCM; (**B–E**) Marker of fibrosis in human biopsies of HOCM patients and healthy controls. n = 6–10.

To investigate the correlation of c-KIT levels directly in the myocardium, we performed c-KIT analysis via qPCR in human myectomies of patients with HOCM and healthy controls (Fig. 4A). Here, a reverse relationship in comparison to serum was detected suggesting a reduced secretion of soluble c-Kit by HOCM myocardium. The significantly higher c-KIT levels in left ventricle biopsies of patients with HOCM may indicate a potential involvement in pro-fibrotic or pro-hypertrophic signaling pathways. To follow this hypothesis, we next measured several established fibrosis markers (collagen 1A1, collagen 3, CTGF and MMP2) in human biopsies with qPCR (Fig. 4B–E). The expression of collagen 1A1 and MMP2 were significantly increased in myectomies of HOCM patients compared to healthy controls, whereas collagen 3 and CTGF expression was not different in both groups (Fig. 4B–E).

**Figure 5:**
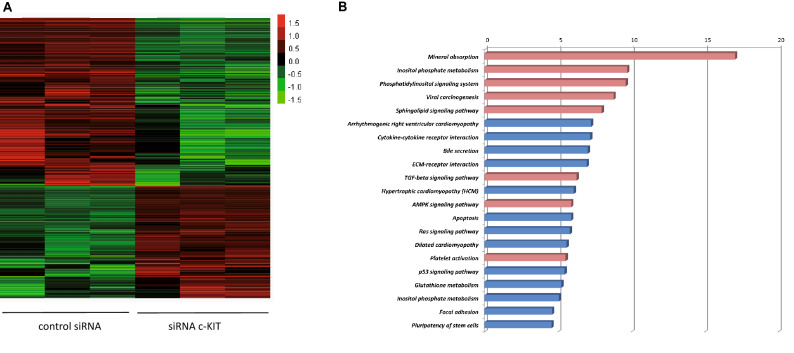
RNA-Sequencing after c-KIT silencing in human cardiac fibroblasts (HCFs). (**A**) Heatmap of RNA Sequencing in human cardiac fibroblasts after treatment with control siRNA or siRNA c-KIT. n = 3 independent experiments. (**B**) Gene set enrichment analysis of 1043 regulated genes with *p* < 0.05 and absolute fold change > 1.5. Functional terms are annotated according to the KEGG molecular functions^40,41^ (red up-regulated, blue downregulated).

Next, we investigated which genes may be associated with c-KIT deregulation in cardiac cells. Therefore we silenced c-KIT expression in human cardiac fibroblasts (HCFs) via siRNA targeting c-KIT or a control siRNA (Suppl. Figure 1) followed by global RNA sequencing. As shown in Fig. 5A we found 1043 deregulated genes after c-KIT silencing. This data set was used for further functional enrichment analysis using David ontology server (Fig. 5B), as described^27^. Several biological processes associated with fibrosis as well as with cardiomyopathies in particular with HCM could be determined. Appropriate to the known data on the cardiometabolic changes in HCM we detected 417 up-regulated and 626 down-regulated genes (*p* < 0.05) in human cardiac fibroblasts after silencing of c-KIT. Fibrotic genes from the TGF-ß signaling pathway as well as genes of inositol phosphate and sphingolipid signaling pathways were up-regulated after c-KIT silencing fitting into the concept of c-KIT being involved in cardiometabolic processes in HCM. Silencing of c-KIT led to the down-regulation of several cardiomyopathic genes, especially of hypertrophic cardiomyopathy as well as of dilated and arrhythmogenic right ventricular cardiomyopathy, revealing a positive effect of c-KIT silencing on several disease entities.

## Discussion

The present study identified c-KIT as a novel protein biomarker for patients with hypertrophic cardiomyopathy and revealed a novel role for c-KIT to be directly involved in cardiac remodeling processes in HCM patients. Serum c-KIT concentrations are negatively correlated with the presence of HCM and positively correlated with echocardiographic parameter LVEDD in the HOCM cohort providing a further distinguishing feature between the patient groups of HOCM and HNCM. Additionally, in fibrotic tissues of human myectomies increased c-KIT levels correlated with higher markers of cardiac fibrosis in the HCM group compared to controls suggesting a possible link to a cardiac fibrosis already known to be associated with HCM. These data were also supported by global RNA sequencing results from human cardiac fibroblasts after c-KIT silencing revealing the involvement of fibrotic and cardiometabolic pathways. The RNA-Seq data give insights into cellular processes and identify strongly regulated pathways, e.g. inositol phosphate pathway involved into the modulation of cellular calcium release known to be affected in HCM as well as sphingolipid signaling pathway regulating several signal transduction processes including cell proliferation, differentiation and apoptosis. Additionally, silencing of c-KIT had a significant effect on the down-regulation of genes of hypertrophic, dilated and arrhythmogenic right ventricular cardiomyopathies.

c-KIT and it’s ligand hematopoietic cytokine stem cell factor (SCF) are essential during the embryonic development and their absence leads to intrauterine or perinatal death due to a severe macrocytic anemia^17^. Binding of SCF to c-KIT leads, via dimerization and phosphorylation, to the activation of the receptor. Activated c-KIT serves as binding site for multiple kinases which in turn activate further pathways. c-KIT tyrosine kinase signaling is involved in several cellular functions such as cell proliferation and survival and plays a decisive role inter alia in hematopoiesis and melanogenesis. c-KIT acts as proto-oncogene in tumors and pharmacological inhibitors of the c-KIT axis are used for therapeutic interventions^28^. Besides the oncologic importance of c-KIT signaling, few studies exist on the importance of c-KIT in the pulmonary and cardiovascular field. Ding et al.^26^ reported about the role of SCF-c-KIT axis in bleomycin induced pulmonary fibrosis (IPF) and showed that expression of c-KIT in fibroblasts from patients with IPF was significantly higher than in those of control patients and there were more fibrosis markers, e.g. TGF-ß in the IPF group. In addition, the authors showed that fibroblasts were the main source of SCF in IPF mice in comparison to the controls and had a chemotactic effect on migration of bone marrow-derived cells to the injured lungs with subsequent paracrine activation of fibroblasts.

c-KIT signaling was also described to be involved in the overexpression of nerve growth factor, which was shown to exert several beneficial effects during injury and repair after myocardial infarction^29^. Moreover, Wang et al. showed that c-KIT signaling contributes to the neointimal formation after arterial injury in mice by upregulation of c-KIT ligand SCF and postulated that inhibition of c-KIT has vasculoprotective effects^30^.

Soluble c-KIT has been known to be released by hematopoietic, mast and endothelial cells^31–33^ and circulates in human serum in concentrations considerably higher than those of circulating SCF. Wypych and colleagues showed that circulating c-KIT binds SCF and blocks SCF-induced cell proliferation^33^ thus acting as an internal inhibitor of SCF and in turn of membrane bound c-KIT in cells. In line with these findings, in our study the levels of c-KIT in serum was significant lower in HCM patients compared to healthy persons without structural heart disease. According to Wypych et al., lower circulating c-KIT levels are associated with more cell proliferation and in case of HCM possibly with more fibrosis. Recently, Sukhacheva et al. examined the resident cardiac stem cells in myectomies of 40 HOCM patients and revealed that in 82,5% of samples c-KIT positive cells could be detected^34^. These myectomies contained increased density of the connective tissue, moderate hypertrophy of the cardiomyocytes and increased markers of heart failure^34^. In the present study we detected c-KIT in myectomies not only of HOCM patients but also in control biopsies and were able to show higher levels of c-KIT in HOCM group than in controls. The extent of cardiac fibrosis in different cardiomyopathies, e.g. HCM, is clinically relevant for the prognosis and risk assessment of ICD implantation due to the potential burden of sudden cardiac death. To correlate our data with a possible role of c-KIT in fibrosis, we measured several fibrosis markers in myectomies and detected elevated levels of pro-fibrotic factors such as Collagen 1A1 and MMP2 in HOCM biopsies compared to healthy samples. In addition to the levels of c-KIT in cardiac tissue, levels of c-KIT in serum of healthy and HCM patients were also measured. Here, we found that c-KIT levels were significant lower in HCM patients than in healthy controls and significantly correlated with echocardiographic parameter of left ventricular end-diastolic diameter in HOCM patients. c-KIT may thus serve as an indicator of HOCM severity in patients. Additionally our in vitro experiments in c-KIT silenced human cardiac fibroblasts and subsequent *in-silico* pathway analysis of RNA Sequencing data revealed that c-KIT is involved in several biological processes associated with fibrosis and in particular with HCM. As cardiac fibrosis is known to correlate with the risk of sudden cardiac death in HCM patients, effects of myocardial c-kit modulation should be investigated in further in vitro heart models (e.g. myocardial slices) and in vivo studies. Our medium-size study should therefore be supported by further studies with larger patient populations to examine the interaction between c-KIT and clinical severity of HCM and detailed molecular mechanisms to develop new therapeutic strategies for HCM patients and to alleviate clinical decision making in these patients.

In conclusion, the present data identified c-KIT as a novel serum and tissue biomarker differentiating between patients with HCM and healthy population. Such biomarker-assisted approach could be added to the current knowledge understanding fibrotic signaling within HCM.

## Materials and methods

### Patient data

Patients with HNCM/HOCM were enrolled at the Special Outpatient Clinic for HCM, Department of Cardiology and Angiology (Hannover Medical School). Blood from healthy controls was obtained from the Hannover Medical School blood donation service. Human biopsies were obtained from patients with HOCM who have undergone myocardial surgery at the Hannover Medical School or from non-transplanted donor hearts (accident victims; Sydney Heart Bank, Australia) as controls. Written informed consents were obtained from all patients before surgery. The study was approved by the local ethic committee of Hannover Medical School (No. 507/09).

The diagnosis of HCM was based on the recent European guidelines for the diagnosis and management of hypertrophic cardiomyopathies^2^ and included presence of a hypertrophic cardiac septum (≥ 15 mm) or combined presence of a hypertrophic cardiac septum (≥ 13 mm) and positive family history and/or ECG abnormalities. HOCM was defined by a left ventricular outflow tract gradient ≥ 30 mmHg to differentiate between non-obstructive and obstructive form.

All methods were carried out in accordance with relevant guidelines^2^ and regulations.

### Measurement of biomarkers

Serum levels of 92 proteins related to cardiometabolic processes were analyzed with multiplex proximity extension assay of the OLINK platform (OLINK Target 96 Cardiometabolic Panel, Olink Proteomics, Uppsala, Sweden), as described before^35–37^. Proximity extension assay (PEA) is based on pairs of antibodies linked to oligonucleotides which bind to their target proteins in the sample. The quantity of the target protein is measured by qPCR. The protein levels are expressed with normalized protein expression (NPX) values, OLINK Proteomics’ arbitrary unit on log2 scale. Detailed information could be found at manufacturer’s website www.olink.com/resources-support/document-download-center.

### Cell culture

Human cardiac fibroblasts (HCFs) were purchased from Promocell and cultured in fibroblast basal medium FBM-3 supplemented with 10% fetal bovine serum (FBS), supplements (Promocell), 100 μg/ml penicillin and 100 μg/ml streptomycin under standard cell culture conditions (37 °C, 5% CO2).

### Transfection experiments

As described before^38^, transient liposomal transfection of cells was performed at a confluence of 60–70% 1 day after seeding. Final concentration of oligonucleotides was 100 nM for siRNA. Oligonucleotides that were used for transfection experiments were as following: siRNA c-KIT (Santa Cruz sc-29225), control siRNA (Santa Cruz sc-37007). siRNAs or control siRNAs and Lipofectamine 2000 (Invitrogen) were mixed separately with Opti-MEM I media (Invitrogen) and incubated for 5 min, then mixed together and incubated again for 20 min. For the transfection reaction serum-free conditions were applied and after 4 h medium was renewed. After 48 h RNA was isolated for RNA sequencing.

### Processing of patient blood

Serum blood samples were collected and centrifuged at 2000 × *g* for 10 min at room temperature. After separation of corpuscular components, the liquid supernatant was stored at − 80 °C in RNase/DNase free tubes until the use for further analysis.

### RNA isolation

Total RNA of tissues (human myectomies) and cultured cardiac fibroblasts (HCFs) was isolated using miRNeasy Mini Kit (Qiagen) or TriFast method (Peqlab) according to the manufacturer’s instructions. The RNA-concentration was measured at 260 nm and 280 nm and the ratio was calculated using the Synergy HT multi-mode Reader (Biotek).

### Reverse transcription and real-time PCR of c-KIT

Isolated RNA was transcribed to complementary DNA (cDNA) using cDNA Synthesis Kit (Biozym) according to manufacturer’s manuals. ß-Actin was amplified as a control. mRNA expression of ß-Actin and c-KIT was analyzed by SYBR Green method using primers for ß-Actin (see table below) and c-KIT. Two Primers for human c-KIT were purchased (Quantitect primer set, Qiagen QT00080409 and QT01679993). The qPCR was carried out in a 384-well plate using the ViiA 7 Real-Time PCR System or QuantStudio 7 Flex System (Thermo Fisher Scientific).

Following primers were used in this study:

**Table:** 

ß-Actin forward	5′ CCTCGCCTTTGCCGATCC3'
ß-Actin reverse	5′ CTTCTGACCCATGCCCACC3'

### ELISA assay for protein analysis

Proteins were analyzed applying ELISA kit systems for c-KIT (Sigma-Aldrich, #RAB0731), IL-7R (Aviva System Biology, #OKEH02803), PCOLCE (Sigma-Aldrich, #RAB1627) and IGLC2 (Aviva System Biology, #OKCA01304) according to manufacturer´s instructions. In brief, 100 µl of each standard and serum sample were incubated in appropriate wells overnight at 4 °C with gentle shaking. Then, the solution was discarded and washed 4 times with wash solution. Afterwards 100 µl of prepared biotinylated detection antibody were added to each well and incubated for 1 h at room temperature with gentle shaking. Again, the solution war discarded, washed 4 times and incubated with 100 µl of prepared HRP-Streptavidin solution for 45 min at room temperature with gentle shaking. After a further washing step 100 µl of ELISA Colorimetric TMB reagent was added to each well, incubated for 30 min at room temperature in the dark with gentle shaking. Finally, 50 µl of stop solution were added to each well and the absorbance was immediately measured at 450 nm.

### RNA Sequencing

#### Library generation, quality control, and quantification

As described before^39^, the following procedures were performed. 300 ng of total RNA per sample were utilized as input for mRNA enrichment procedure with ‘NEBNext Poly(A) mRNA Magnetic Isolation Module’ (E7490L; New England Biolabs) followed by stranded cDNA library generation using ‘NEBNext Ultra II Directional RNA Library Prep Kit for Illumina’ (E7760L; New England Biolabs). All steps were performed as recommended in user manualE7760 (Version 1.0_02-2017; NEB) except that all reactions were downscaled to 2/3 of initial volumes. Furthermore, one additional purification step was introduced at the end of the standard procedure, using 1x ‘Agencourt AMPure XP Beads’ (#A63881; Beckman Coulter, Inc.).

cDNA libraries were barcoded by dual indexing approach, using ‘NEBNext Multiplex Oligos for Illumina—96 Unique Dual Index Primer Pairs’ (6440S; New England Biolabs). All generated cDNA libraries were amplified with 8 cycles of final pcr.

Fragment length distribution of individual libraries was monitored using ‘Bioanalyzer High Sensitivity DNA Assay’ (5067–4626; Agilent Technologies). Quantification of libraries was performed by use of the ‘Qubit dsDNA HS Assay Kit’ (Q32854; ThermoFisher Scientific).

#### Library denaturation and Sequencing run

Equal molar amounts of six individually barcoded libraries were pooled. Accordingly, each analyzed library constitutes 16.7% of overall flowcell capacity. The library pool was denatured with NaOH and was finally diluted to 1.8 pM according to the Denature and Dilute Libraries Guide (Document # 15,048,776 v02; Illumina). 1.3 ml of denatured pool was loaded on an Illumina NextSeq 550 sequencer using a Mid Output Flowcell for 2 × 75 bp paired-end reads (20,024,904; Illumina). Sequencing was performed with the following settings: Sequence read 1 with 76 bases; Index reads 1 and 2 with 8 bases each.

#### BCL to FASTQ conversion

BCL files were converted to FASTQ files using bcl2fastq Conversion Software version v2.20.0.422 (Illumina).

#### Raw data processing and quality control

Raw data processing was conducted by use of nfcore/rnaseq (version 1.3) which is a bioinformatics best-practice analysis pipeline used for RNA sequencing data at the National Genomics Infrastructure at SciLifeLab Stockholm, Sweden. The pipeline uses Nextflow, a bioinformatics workflow tool. It pre-processes raw data from FastQ inputs, aligns the reads and performs extensive quality-control on the results. The genome reference and annotation data were taken from GENCODE.org (Homo sapiens; GRCh38; release 29).

#### Normalization and differential expression analysis

Normalization and differential expression analysis was performed with DESeq2 (Galaxy Tool Version 2.11.40.2) with default settings except for “Output normalized counts table” was set to “True”.

#### Normalization and differential expression analysis (without outlier filtering)

Normalization and differential expression analysis was performed with DESeq2 (Galaxy Tool Version 2.11.40.2) with default settings except for “Output normalized counts table”, “Turn off outliers filtering”, and “Turn off independent filtering”, all of which were set to “True”.

### Gene set enrichment analysis

List of the 1043 significant genes (*p* value < 0.05) were submitted to DAVID functional annotation tool^27^ for gene set enrichment analysis. KEGG pathway was used as annotation resource^40,41^.

### Statistical analysis

The statistical software package R version 3.5.2 (www.r-project.org) was used for statistical analyses. The characteristics of the studied population were summarized through standard descriptive statistics. Data are presented as the median (25th percentile, P25 – 75th percentile, P75) for continuous variables and as frequency (percentage) for categorical variables. The demographic, clinical, echocardiographic and biochemical characteristics were compared between groups Mann–Whitney U test for continuous variables and Chi-squared test or Fisher's exact test for categorical variables. Spearman’s rho coefficient was used to assess the correlation between continuous variables. Logistic regression was used to assess the relationship between c-Kit and HCM. To establish whether the associations could be influenced by confounding factors, the association was adjusted by age and sex. The results are presented as odds ratios (OR) and 95% confidence intervals (CI). Receiver operating characteristic (ROC) curves were constructed to estimate the area under the ROC curve. Data are presented as the C-index and 95% CI. The two-tailed significance level was set at < 0.05.

## Supplementary Information

Supplementary Information.

## Data Availability

All data generated or analyzed during this study are included in this published article and its Supplementary Information files.

## References

[1] Maron BJ (2018). Clinical course and management of hypertrophic cardiomyopathy. N. Engl. J. Med..

[2] Authors/Task Force membersmembers, A. F. *et al.* 2014 ESC guidelines on diagnosis and management of hypertrophic cardiomyopathy: the task force for the diagnosis and management of hypertrophic cardiomyopathy of the European Society of Cardiology (ESC). *Eur. Heart J.* **35**, 2733–2779 (2014). 10.1093/eurheartj/ehu284 25173338

[3] Sonnenschein K (2019). Serum circular RNAs act as blood-based biomarkers for hypertrophic obstructive cardiomyopathy. Sci. Rep..

[4] Kitow J (2016). (2016) Mitochondrial long noncoding RNAs as blood based biomarkers for cardiac remodeling in patients with hypertrophic cardiomyopathy. Am. J. Physiol. Hear. Circ. Physiol..

[5] Derda AA (2015). Blood-based microRNA signatures differentiate various forms of cardiac hypertrophy. Int. J. Cardiol..

[6] Huang, D. *et al.* MicroRNA-221 is a potential biomarker of myocardial hypertrophy and fibrosis in hypertrophic obstructive cardiomyopathy. *Biosci. Rep.* **40**(1), BSR20191234 (2020). 10.1042/BSR20191234 PMC6954366 31868204

[7] Roncarati R (2014). Circulating miR-29a, among other up-regulated microRNAs, is the only biomarker for both hypertrophy and fibrosis in patients with hypertrophic cardiomyopathy. J. Am. Coll. Cardiol..

[8] Magnusson P (2020). Positron emission tomography ((15)O-water, (11)C-acetate, (11)C-HED) risk markers and nonsustained ventricular tachycardia in hypertrophic cardiomyopathy. Int. J. Cardiol. Hear. Vasc..

[9] Ricci F (2019). Pulmonary blood volume index as a quantitative biomarker of haemodynamic congestion in hypertrophic cardiomyopathy. Eur. Heart J. Cardiovasc. Imaging.

[10] Bégué C (2020). Mid-regional proatrial natriuretic peptide for predicting prognosis in hypertrophic cardiomyopathy. Heart.

[11] Coats CJ (2018). Proteomic analysis of the myocardium in hypertrophic obstructive cardiomyopathy. Circ. Genomic Precis. Med..

[12] van der Velden J (2018). Metabolic changes in hypertrophic cardiomyopathies: scientific update from the Working Group of myocardial function of the European Society of Cardiology. Cardiovasc. Res..

[13] Toepfer CN (2020). Myosin sequestration regulates sarcomere function, cardiomyocyte energetics, and metabolism, informing the pathogenesis of hypertrophic cardiomyopathy. Circulation.

[14] Ashrafian H, Redwood C, Blair E, Watkins H (2003). Hypertrophic cardiomyopathy:a paradigm for myocardial energy depletion. Trends Genet..

[15] Cruz AC (2004). Tumor necrosis factor-alpha-converting enzyme controls surface expression of c-Kit and survival of embryonic stem cell-derived mast cells. J. Biol. Chem..

[16] Cardoso HJ, Figueira MI, Socorro S (2017). The stem cell factor (SCF)/c-KIT signalling in testis and prostate cancer. J. Cell Commun. Signal..

[17] Broudy VC (1997). Stem cell factor and hematopoiesis. Blood.

[18] Tang J (2020). Targeted sequencing reveals the mutational landscape responsible for sorafenib therapy in advanced hepatocellular carcinoma. Theranostics.

[19] Fujimoto S (2018). A novel theranostic combination of near-infrared fluorescence imaging and laser irradiation targeting c-KIT for gastrointestinal stromal tumors. Theranostics.

[20] Guo J (2011). Phase II, open-label, single-arm trial of imatinib mesylate in patients with metastatic melanoma harboring c-Kit mutation or amplification. J. Clin. Oncol. Off. J. Am. Soc. Clin. Oncol..

[21] Kim J-O (2020). Development and characterization of a fully human antibody targeting SCF/c-kit signaling. Int. J. Biol. Macromol..

[22] Koch C, Trojan J (2015). Established and potential predictive biomarkers in gastrointestinal cancer–c-Kit, Her2, ras and beyond. Digestion.

[23] Deprimo SE (2009). Circulating levels of soluble KIT serve as a biomarker for clinical outcome in gastrointestinal stromal tumor patients receiving sunitinib following imatinib failure. Clin. cancer Res. Off J. Am. Assoc. Cancer Res..

[24] Kalathil SG, Wang K, Hutson A, Iyer R, Thanavala Y (2020). Tivozanib mediated inhibition of c-Kit/SCF signaling on Tregs and MDSCs and reversal of tumor induced immune suppression correlates with survival of HCC patients. Oncoimmunology.

[25] Tarlock K (2019). Functional Properties of KIT mutations are associated with differential clinical outcomes and response to targeted therapeutics in CBF acute myeloid leukemia. Clin. Cancer Res. Off J. Am. Assoc. Cancer Res..

[26] Ding L (2013). Essential role of stem cell factor-c-Kit signalling pathway in bleomycin-induced pulmonary fibrosis. J. Pathol..

[27] Huang DW, Sherman BT, Lempicki RA (2009). Systematic and integrative analysis of large gene lists using DAVID bioinformatics resources. Nat. Protoc..

[28] Lamb Y (2019). N. pexidartinib: first approval. Drugs.

[29] Meloni M (2010). Nerve growth factor promotes cardiac repair following myocardial infarction. Circ. Res..

[30] Wang C-H (2006). Stem cell factor deficiency is vasculoprotective: unraveling a new therapeutic potential of imatinib mesylate. Circ. Res..

[31] Broudy VC (1994). Human umbilical vein endothelial cells display high-affinity c-kit receptors and produce a soluble form of the c-kit receptor. Blood.

[32] Turner AM (1995). Identification and characterization of a soluble c-kit receptor produced by human hematopoietic cell lines. Blood.

[33] Wypych J (1995). Soluble kit receptor in human serum. Blood.

[34] Sukhacheva TV (2012). Resident stem cells in the myocardium of patients with obstructive hypertrophic cardiomyopathy. Bull. Exp. Biol. Med..

[35] Assarsson E (2014). Homogenous 96-plex PEA immunoassay exhibiting high sensitivity, specificity, and excellent scalability. PLoS ONE.

[36] Solier C, Langen H (2014). Antibody-based proteomics and biomarker research—current status and limitations. Proteomics.

[37] Bouwens E (2020). Circulating biomarkers of cell adhesion predict clinical outcome in patients with chronic heart failure. J. Clin. Med..

[38] Sonnenschein K (2019). Therapeutic modulation of RNA-binding protein Rbm38 facilitates re-endothelialization after arterial injury. Cardiovasc. Res..

[39] Kenneweg F (2019). Long noncoding RNA-enriched vesicles secreted by hypoxic cardiomyocytes drive cardiac fibrosis. Mol. Ther. Nucleic Acids.

[40] Kanehisa M, Goto S (2000). KEGG: kyoto encyclopedia of genes and genomes. Nucleic Acids Res..

[41] Kanehisa M, Furumichi M, Sato Y, Ishiguro-Watanabe M, Tanabe M (2020). KEGG: integrating viruses and cellular organisms. Nucleic Acids Res..

