# Supplementary material for: Blood-based protein profiling identifies serum protein c-KIT as a novel biomarker for hypertrophic cardiomyopathy
Source: Sci Rep. 2021 Jan 19;11:1755. doi: 10.1038/s41598-020-80868-z (PMC7815737; doi:10.1038/s41598-020-80868-z)
Supplement: Supplementary file 1 — Supplementary Information. [file 41598_2020_80868_MOESM1_ESM.pdf]

SUPPLEMENTARY INFORMATION

Blood-based protein profiling identifies serum protein c-KIT as a novel biomarker for hypertrophic cardiomyopathy

Short title: c-KIT in hypertrophic cardiomyopathy

Kristina Sonnenschein[1,2], Jan Fiedler[1], David de Gonzalo-Calvo[1,3,4], Ke Xiao[1], Angelika Pfanne[1], Annette Just[1], Carolin Zwadlo[2], Samira Soltani[2], Udo Bavendiek[2], Theresia Kraft[5], Cristobal Dos Remedios[6], Serghei Cebotari[7], Johann Bauersachs[2,8], Thomas Thum[1,8,9]

[1] Institute of Molecular and Translational Therapeutic Strategies (IMTTS), Hannover Medical School, Hannover, Germany

[2] Department of Cardiology and Angiology, Hannover Medical School, Hannover, Germany

[3] CIBER of Respiratory Diseases (CIBERES), Institute of Health Carlos III, Av. de Monforte de Lemos, 5, 28029 Madrid, Spain

[4] Translational Research in Respiratory Medicine, University Hospital Arnau de Vilanova and Santa Maria, IRBLleida, Av. Alcalde Rovira Roure, 80, 25198 Lleida, Spain.

[5] Institute of Molecular and Cell Physiology, Hannover Medical School, Hannover, Germany

[6] Anatomy and Histology, School of Medical Sciences, Bosch Institute, University of Sydney, Australia

[7] Department of Cardiac, Thoracic, Transplantation, and Vascular Surgery, Hannover Medical School, Hannover, Germany

[8] REBIRTH Center for Translational Regenerative Medicine, Hannover Medical School, Hannover, Germany

[9] Fraunhofer Institute of Toxicology and Experimental Medicine, Hannover, Germany

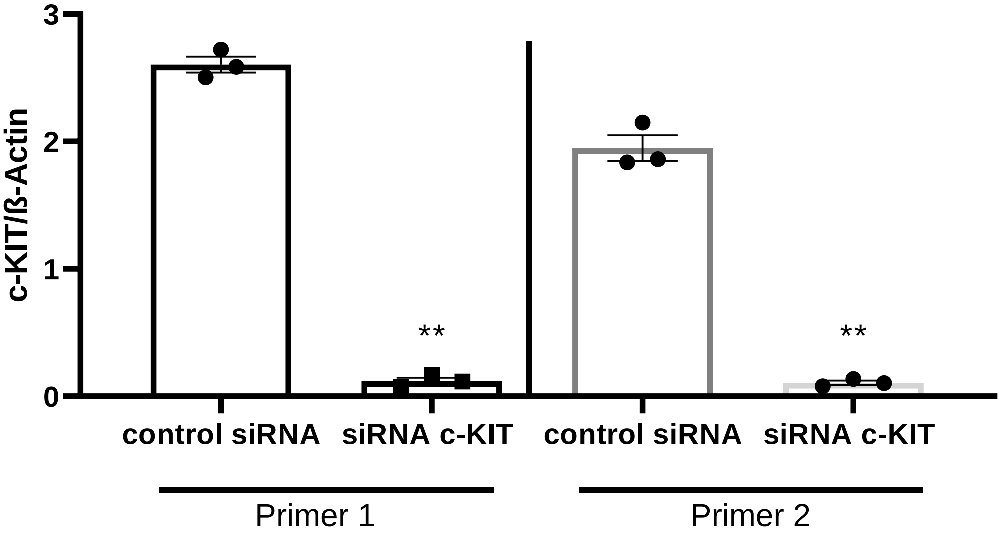

Supplemental Figure 1. Validation of the expression of c-KIT in HCFs after transfection with 100 nM siRNA c-KIT or control siRNA using qPCR. n = 3 independent experiments with technical triplicates. ** p< 0.0001. Statistical significance was calculated by unpaired student's t-test.
